# Analgesia during Parturition in Domestic Animals: Perspectives and Controversies on Its Use

**DOI:** 10.3390/ani12192686

**Published:** 2022-10-06

**Authors:** Daniel Mota-Rojas, Antonio Velarde, Míriam Marcet-Rius, Agustín Orihuela, Andrea Bragaglio, Ismael Hernández-Ávalos, Alejandro Casas-Alvarado, Adriana Domínguez-Oliva, Alexandra L. Whittaker

**Affiliations:** 1 Neurophysiology, Behavior and Animal Welfare Assessment, DPAA, Universidad Autónoma Metropolitana, (UAM), Mexico City 04960, Mexico; 2 Institute of Agrifood Research and Technology (IRTA), Animal Welfare Program, Veinat Sies S-N, 17121 Monells, Spain; 3 Animal Behaviour and Welfare Department, IRSEA (Research Institute in Semiochemistry and Applied Ethology), Quartier Salignan, 84400 Apt, France; 4 Facultad de Ciencias Agropecuarias, Universidad Autónoma del Estado de Morelos, Cuernavaca 62209, Mexico; 5 Department of Veterinary Medicine, University of Bari A. Moro, 70010 Valenzano, Italy; 6 Clinical Pharmacology and Veterinary Anesthesia, Facultad de Estudios Superiores Cuautitlán, Universidad Nacional Autónoma de México (UNAM), Mexico 54714, Mexico; 7 School of Animal and Veterinary Sciences, Roseworthy Campus, University of Adelaide, Roseworthy, SA 5116, Australia

**Keywords:** pain, labor pain, calving, farrowing, analgesics

## Abstract

**Simple Summary:**

Although the pain associated with parturition performs the primary physiological function of maintaining frequent, strong myometrial contractions, its biological consequences can affect the health of both mother and fetus. Whilst analgesic therapy may be recommended to avoid pain, the evidence indicates that the mechanisms of some analgesic drugs can interfere with the biological process of labor. Opioids and non-steroidal anti-inflammatory drugs have been shown to indirectly inhibit myometrial contractions by decreasing oxytocin secretion, while local analgesics decrease the number of contractions, although its intensity increases, improving maternal performance. This article analyzes the physiological role of pain during labor and the use and efficacy of opioids, non-steroidal anti-inflammatory drugs, and local analgesics in treatments to manage parturition in domestic mammals.

**Abstract:**

This article analyzes the physiological role of pain during parturition in domestic animals, discusses the controversies surrounding the use of opioids, non-steroidal anti-inflammatory drugs (NSAIDs), and local analgesics as treatments during labor, and presents the advantages and disadvantages for mother and offspring. Labor is a potentially stressful and painful event, due to the contractions that promote expulsion of the fetus. During labor, neurotransmitters such as the prostaglandins contribute to the sensitization of oxytocin receptors in the myometrium and the activation of nociceptive fibers, thus supporting the physiological role of pain. Endogenously, the body secretes opioid peptides that modulate harmful stimuli and, at the same time, can inhibit oxytocin’s action in the myometrium. Treating pain during the different stages of parturition is an option that can help prevent such consequences as tachycardia, changes in breathing patterns, and respiratory acidosis, all of which can harm the wellbeing of offspring. However, studies have found that some analgesics can promote myometrial contractility, increase expulsion time, affect fetal circulation, and alter mother–offspring recognition due to hypnotic effects. Other data, however, indicate that reducing the number of uterine contractions with analgesics increases their potency, thus improving maternal performance. Managing pain during labor requires understanding the tocolytic properties of analgesics and their advantages in preventing the consequences of pain.

## 1. Introduction

It is well established that the avoidance of pain is beneficial for animal welfare. In order to achieve this, adequate, timely management techniques are required to ensure animal wellbeing and mitigate the sensory and physiological consequences of pain [1,2]. In domestic animals, parturition is an event that normally involves pain, but controversy surrounds the use of analgesics during labor because of conflicting reports as to their benefits in controlling pain, their ability to maintain productive functions and adequate maternal performance [3], their tocolytic effects, and the inhibition of uterine contractions that may prolong labor and affect fetal circulation and/or the dam’s recognition of her newborn(s) [4,5,6]. A further challenge is in the practical implementation of analgesic protocols, since animals may show few signs that parturition is imminent and may give birth away from stockpeople who can intervene. 

Eutocic parturition is considered a stressful and painful event, mainly because of the frequent, strong uterine contractions that occur during this process [7]. The physiological consequences of pain during the various phases of parturition derive from chemical mediators such as prostaglandins (PG) and cortisol, which sensitize oxytocin receptors in the myometrium to promote contractions [8] but can also trigger autonomic responses such as tachycardia, hypertension, changes in breathing patterns, and a state of acidosis [9]. 

Pain is also involved in the maintenance of myometrial contractions via positive feedback [10] in association with the release of endogenous opioid peptides (EOP) that decrease oxytocin sensitization and the frequency of uterine contractions, with the consequence that an eutocic delivery could be converted into a dystocic one [11,12]. In contrast, some studies have shown that a decrease in the number of contractions as a result of analgesic administration is not necessarily associated with complications during labor, but may actually increase the strength of contractions and lessen pain-related consequences [13,14,15]. Given this controversy, in which some authors sustain that the role of pain during parturition is limited [16], while others argue that pain is an essential element of the normal course of labor [10], we set out to analyze the physiological role of pain during parturition and discuss the usefulness of local anesthetics, non-steroidal anti-inflammatory drugs (NSAIDs), and opioids as treatments during labor in domestic animals, as well as the potential advantages and disadvantages of their use for both mother and offspring.

## 2. Controversy on the Participation of Pain during Parturition 

**Figure 1:**
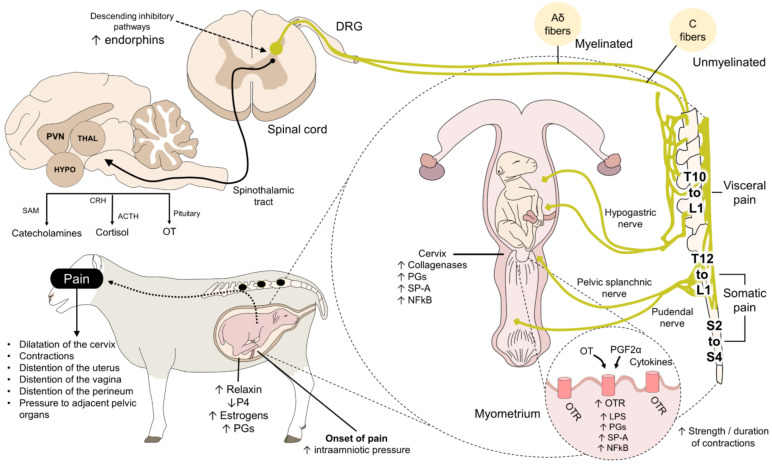
Neurobiology of pain during parturition. With the onset of labor and its clinical signs, such as cervical dilation, myometrial contractions, uterine distention, and increased concentrations of PG, estrogen, collagenase, and other mediators, a nociceptive response is detected by Aδ and C fibers. These neurons transmit sensations of visceral and somatic pain to the dorsal horn of the spinal cord, where interneurons modulate and project to supraspinal centers to trigger the conscious perception of pain. Brain areas such as the HYPO and PVN are responsible for generating physiological responses that trigger secretion of catecholamines, glucocorticoids (cortisol), and OT. OT is the main hormone associated with the onset and maintenance of labor. Its secretion, together with PGF2α and cytokines, activates OTR to increase the strength, frequency, amplitude, and duration of myometrial contractions. ACTH: adrenocorticotropic hormone; CRH: corticotropin-releasing hormone; DRG: dorsal root ganglion; HYPO: hypothalamus; LPS: lipopolysaccharides; NFkB: nuclear factor-kB; OT: oxytocin; OTR: oxytocin receptors; P4: progesterone; PG: prostaglandins; PVN: paraventricular nucleus; SAM: sympathetic-adrenomedullary axis; SP-A: surfactant protein A; TAL: thalamus.

Mainau and Manteca [16] established that parturition is a physiological stage in which the perception of acute pain results from uterine contractions, the expulsion of the fetus, and inflammation of the uterine tract. Uterine contractions are necessary, especially during the expulsion phase, when they increase in strength and duration, increasing pain intensity (Figure 1) [17,18]. 

Martínez-Burnes et al. [7] described the physiological role of pain that, together with the degree of fetal maturity, transform the uterus from a quiescent organ to an active one with an abundant presence of oxytocin receptors and proteins such as connexin 43 (Cx43), the elements that cause sustained, synchronic contractions of the myometrium [19,20]. Reports on mammals suggest that the increase in binding sites in the uterus occurs 24 h before the onset of parturition, and that their number increases over 300-fold once the phase of parturition begins [21]. 

Secretion of oxytocin is required for contractions to occur. Experimental models of rodents have shown that pain has a positive effect on oxytocin secretion [22]. In rodents, oxytocin neurons produce a series of synchronic bursts of this hormone that later act on myometrial oxytocin receptors, while oxytocin neurons generate signals at the level of the spinal cord and vagal nerve directed to α2 noradrenergic cells. This creates a self-sustaining positive feedback cycle that culminates with the release of oxytocin and maintains contractions. The cycle is known as the Ferguson reflex [10,23,24,25,26]. In addition, increased noradrenaline concentrations in the supraoptic nucleus (SON) and magnocellular region of the paraventricular nucleus (where the α1-adrenoreceptors are located) activate oxytocin neurons during the phases of parturition, promoting oxytocin synthesis and secretion [27].

Oxytocin is associated with an analgesic effect, achieved by changing the permeability of the chloride ion in GABAergic neurons [28]. The peripheral, spinal, and supraspinal analgesic action of oxytocin is also linked to hyperpolarization, mediated by a Ca2+-dependent mechanism [29] and by the interaction with transient potential vanilloid 1 receptors (TRPV1). Subcutaneous administration of oxytocin reduces capsaicin-induced nociception in rats (*Rattus norvegicus*) and mice (*Mus musculus*), due to a desensitization effect on the ion channels [30,31]. The function of pain, oxytocin secretion and its potential analgesic role are, however, thought to be limited when an acute process generates an attenuation response by the hypothalamus–pituitary–adrenal axis (HPA). Activation of the HPA releases endogenous opioid peptides (EOP) such as dynorphin and β-endorphins that inhibit oxytocin secretion by occupying κ receptors in the neuronal terminals of the anterior pituitary. As a direct consequence, this reduces secretion of the adrenocorticotropic hormone (ACTH), causing a decrease in the number and intensity of uterine contractions [11,32,33,34,35]. 

Another consequence of labor pain is the activation of the autonomic nervous system (ANS). This leads to an increase in catecholamine secretion. The increase in plasma concentrations has been considered to have a tocolytic effect on uterine motility that reduces myometrial contractibility by occupying β-adrenergic receptors in the sympathetic nervous system (SNS), and promoting muscular relaxation [36]. Studies in human medicine have demonstrated that β-adrenergic stimulation also reduces the synthesis of PGF2α and PGE2 [36], two mediators that participate in the expulsion of the fetus and placenta [37]. The relaxation of the myometrium and uterine contractions prevention result in prolonged farrowing and increased number of nociceptive signals. Contrarily, the antagonism of β-receptors with (carbazolyl), in the Sabuncu et al. [38] analysis of 150 German Landrace sows at 111 days of gestation, showed a reduction in farrowing time from 4.7 ± 0.3 h to 4.0 ± 0.3 h. However, Bostedt and Rudloff [39] found that the action of catecholamines on the myometrium depends on α and β receptors, since their relative abundance can vary in relation to the reproductive cycle stages. It is well known that α receptors predominate during estrus, while β receptors increase in number as gestation advances, so it can be inferred that catecholamines action and the presence of predominant adrenergic receptors can alter myometrial activity. 

The physiological consequences described in relation to the decrease in myometrial motility have repercussions not only for the condition of the gestating female, but also for the health and vitality of her offspring. In this regard, Olmos-Hernández et al. [40] evaluated the effect of parity number on the presentation of dystocic farrowing in 120 hybrid Yorkshire × Landrance sows. In that study, gilts presented more contractions than sixth-parity sows, greater contraction intensity (12.30 mmHg), and more intrapartum stillbirths. Those results were attributed to the deleterious effect of prolonged, sustained myometrial activity on fetal circulation, which significantly decreased gas exchange within the placenta and produced neonatal hypoxia in the fetus. Later studies confirmed that maternal stress produces hyperactivity of the HPA and the sympathetic–adrenal–medullary system, related to catecholamine release, elevated fetal blood pressure, and poor placenta-uterine perfusion [41,42]. 

The relationship between catecholamines and pain and stress responses during lambing was investigated in a study where 2 h of psychosocial stress was imposed through isolation of the gestating ewes. The response was measured in uterine blood flow that utilized an adrenergic block with labetalol to determine the influence and impact of catecholamines on the fetus. The results showed fetal cortisol levels 8.1 ± 2.1% higher than in the dams, elevated noradrenaline concentrations, and a 22% reduction in uterine blood flow that produced prolonged anaerobic metabolism and fetal hypertension. It is important to note that the adrenergic block impeded the reduction in fetal blood flow, indicating the role that hormones and neurotransmitters play in the health outcomes of fetuses [43]. 

**Figure 2:**
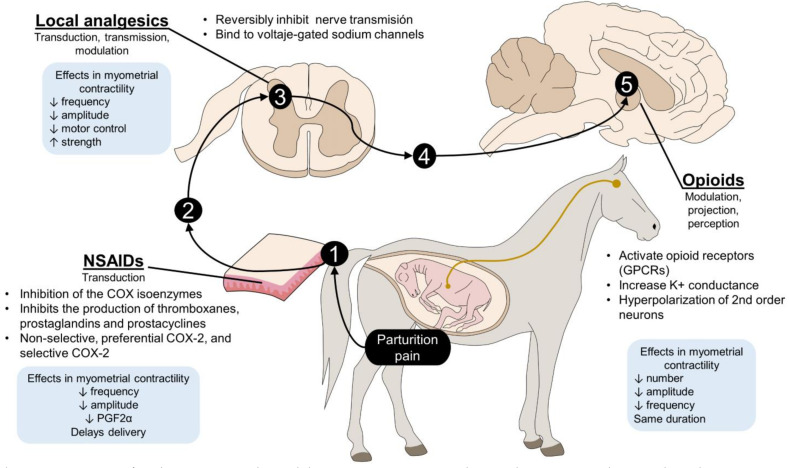
Action site of analgesics commonly used during parturition. Depending on their action mechanism, these drugs intervene at different levels of the nociceptive pathway. The NSAIDs block harmful impulse transduction by inhibiting COX and PG production, while LA can intervene in the first three stages of the nociceptive pathway by blocking voltage-gated sodium channels in the spinal cord. Secondly, opioids are centrally acting analgesics that hyperpolarize neurons to prevent the projection and perception of pain. In general, all three types of drugs can potentially decrease the frequency and amplitude of contractions of the uterus, but this effect depends on the drug and dose administered. 1: transduction; 2: transmission; 3: modulation; 4: projection; 5; perception; COX: enzyme cyclooxygenase. GPCRs: G protein-coupled receptors; PGF2α: prostaglandin 2α.

As Mainau et al. [44] discuss, the occurrence of uncontrolled pain during parturition alters the physiological effect of this condition and triggers a cascade of events ending with the secretion of glucocorticoids (cortisol) that, in conjunction with EOP, inhibit oxytocin release. When concentrations of oxytocin are altered during the expulsion phase, direct or indirect consequences for the fetus can include hypoxia, aggression by the dam, and rejection of the offspring, as well as inhibition of colostrum and milk ejection, increasing pre-weaning mortality. Analgesic therapy of dams during labor might be justified to avoid these problems. However, as we discuss in the following sections, the site and mechanism of action, and adverse effects of the analgesics often employed during birth, must be well understood and evaluated before deciding to apply any analgesic protocol (Figure 2).

## 3. Opioids

**Figure 3:**
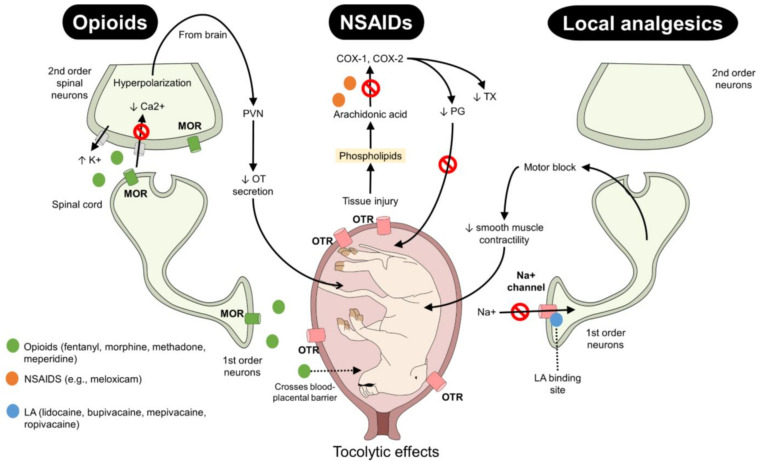
Action mechanisms of opioids, NSAIDs, and local analgesics. During labor, all three types of analgesics exert their action by interacting with various receptors and metabolic pathways. Opioids activate MOR in first- and second-order neurons to prevent Ca2+ influx, increase K conductivity, and hyperpolarize the membrane. NSAIDs block the COX enzyme and production of PGs. Local analgesics block Na+ channels, preventing the transmission of harmful stimuli, but also exert a tocolytic effect by generating a motor blockade that reduces the smooth muscle contractility of the myometrium. Similarly, the decreases in PG production and OT secretion affect the contractility of the uterus. COX: cyclooxygenase; LA: local analgesics; MOR: mu-opioid receptors; NSAIDs: non-steroidal anti-inflammatory drugs; OT: oxytocin; OTR: oxytocin receptors; PVN: paraventricular nucleus.

These analgesics are generally considered superior for control of moderate-to-severe acute pain through agonism to μ, κ, and λ receptors [45]. Their action inhibits the action of G protein-coupled receptors (GPCRs) of cyclic adenosine monophosphate (cAMP), generating an increase in the conductance to K+ that promotes hyperpolarization of the membrane in second-order neurons (Figure 3) [46,47]. Morphine, fentanyl, methadone, and meperidine are the most commonly applied opioids for pain management [7]. However, their effects on parturition are controversial, and may be species dependent.

The analgesic action of morphine at a dosage of 30 μg/10 μL administered with subcutaneous oxytocin was evaluated in 62 gestating albino Sprague Dawley rats based on observing nocifensive behaviors. In the animals that received morphine, the researchers registered a reduction in the number of stretches, a behavior associated with the nociceptive component of uterine contractions, compared to a control group that received an epidural saline solution (frequency of 8 ± 2 vs. 57 ± 12 stretches). The authors did not report significant alterations in mean labor duration, but the rodents treated with the opioid had lower amounts of spinal c-Fos-positive neurons (80 ± 21 vs. 165 ± 17) [48]. C-Fos expression is considered a neuronal marker of nociceptive activation by the spinal afferent fibers that responds to visceral uterine pain during labor in the first 30 min after stimulus onset [49].

Tong et al. [50] determined that intrathecal administration of morphine at infusion rates above 0.035 microg/h administered one day before the onset of parturition, eliminated phasic stretching in 25 primiparous gestating Sprague Dawley rats. By blocking the pain caused by the distension and inflammation of the pelvic viscera, morphine reduced the perception of the harmful stimulus, but showed no adverse effects on the duration of labor, on the presentation of normal activities (e.g., eating, drinking, grooming), or on maternal behaviors such as nest-building, pup-licking, or eating the placenta. The authors thus suggested intrathecal application of morphine as a pharmacological option for treating pain during the birth process. 

In spite of these in vivo studies finding no difference in labor duration with opioid administration, other in vivo and tissue-based studies have shown that contractility of smooth muscle is modified by opioids. Nacitarhan et al. [4], evaluated the cumulative effect of alfentanil, meperidine, and remifentanil on strips of the longitudinal uterine smooth muscle of gestating rats (18–21 days). After inducing contractions with 1 mU/mL of oxytocin, the authors reported that the opioids and local analgesics (LA) such as bupivacaine and ropivacaine significantly reduced myometrial contractions. Similarly, the tocolytic action of morphine and its impact on labor have also been reported by Javadi-Paydar et al. [51], who studied NMRI (Naval Medical Research Institute) mice at 15 days of gestation. Those authors evaluated the effect of a single intraperitoneal (IP) dose of morphine (10 or 20 mg/kg) vs. a double dose (5 or 10 mg/kg) in lipopolysaccharide (LPS)-induced births. The opioid administered in the double dose reduced the incidence of LPS-induced preterm delivery by 50 ± 17.7% compared to the control group, a result that meant an interruption or delay of parturition due to diminished oxytocin secretion [6]. It has been suggested that the myometrial effect of exogenous administration of opium-derived analgesics is related to a mechanism similar to that of the EOP during parturition, since the presence of a large amount of opioid receptors in PVN neurons decreases oxytocin secretion with the expected consequences [27]. For example, morphine increases the rate of the metabolic clearance of oxytocin, which has been shown to reduce uterine contractions by up to 17%, 30–45 min after administration [52]. 

In contrast to the findings in rodents, the use of opioids in humans has been associated with respiratory depression, altered neonatal vitality scores, higher mortality, and problems in mother–offspring interaction due to hypnotic effects [53]. There have also been reports of a tocolytic effect on myometrial activity, suggesting that this could affect maternal performance at birth [54,55,56]. These findings are corroborated by a study in baboons (*Papio anubis*) in their third trimester of gestation (140–150 days). The animals were treated with one 5 mg dose of morphine intravenously (IV) after receiving a bolus injection of oxytocin (500 mU by intravenous infusion) to induce uterine contractions. Morphine increased the metabolic clearance rate by 40%, and significantly reduced the amplitude and frequency of contractions (27 ± 8 mmHg and 3.2 ± 0.5 contractions/15 min, respectively) but did not affect their mean duration [57]. 

The delay in fetus expulsion may have physiological consequences for both, the dam and her neonate, manifested in myometrial dysfunction, fetal asphyxia, stillbirths, maternal and fetal morbidity, and fetal cardiorespiratory depression [58]. The occurrence of these adverse effects is attributed, in some cases, to the pharmacokinetics of the drugs. One example of this was observed in a study of 16 gestating ewes that received a 5 μg/kg-dose of fentanyl by the IV route. The measured transplacental rate of the drug was 77% [59], which is greater than that seen with morphine, likely due to the greater liposolubility of fentanyl. For the neonates, the opioids’ capacity to cross the blood–placental barrier is considered the main cause of respiratory depression, fetal cardiovascular depression, mood depression, inhibition of the sucking reflex, and delayed colostrum intake [6,60]. Butorphanol and pethidine may be particularly problematic for the neonate since these drugs easily cross the blood–placental barrier due to the umbilical vein/maternal vein ratio [61]. The elimination half-life of butorphanol in milk is 36 h, so its administration to the mothers could be considered a risk factor for the safety of the neonate [62], as was reported in newborn colts, where a sedated state [63] with significant respiratory depression was observed. Furthermore, in the offspring, the half-life and distribution volume of this drug were greater (half-life = 2.1 h, VDss = 3.86 L/kg, respectively) than in adults (half-life = 0.74 h, VDss = 1.13 L/kg) [64].

In an effort to prevent negative effects on myometrial contractility and depressor effects in the neonate, some authors have proposed the use of atypical opioids such as tramadol, which work through inhibiting serotonin and norepinephrine reuptake in sensitive fibers [65], as an option that does not alter uterine activity. A study by Yakovleva et al. [66] evaluated tramadol’s effect on uterine contractile activity in chinchillas (*Oryctolagus cuniculus*) at 28 days of gestation. Animals received 1 U of oxytocin by IV to initiate labor, and then 1 mL of tramadol in infusion at a concentration of 5 mg/mL, 10 min later. In that study, tramadol administration did not affect the number of contractions, but increased their amplitude and duration compared to the control group. Their results thus differed from those reported for common opioids such as morphine, fentanyl, and butorphanol [4,50].

In conclusion, even though opioids offer efficacious pain management during parturition, their adverse effects–depressed mental states, reduced oxytocin secretion–hinder mother–offspring interaction in some species and can indirectly affect uterine activity and neonate survival during the first days of life. For these reasons, other therapeutic options are available that do not exert their action at the central level. These include the NSAIDs.

## 4. Non-Steroidal Anti-Inflammatory Drugs

The NSAIDs are a group of drugs widely used in veterinary medicine to manage acute pain [65,67,68]. They work through inhibiting the enzyme cyclooxygenase (COX), which has three isoforms (COX-1, COX-2, COX-3) that modulate the inflammatory response which follows the production of chemical mediators such as PG, thromboxane A, and the prostacyclin [69,70]. However, the use of NSAIDs during parturition is controversial, and some adverse effects on uterine contractions have been attributed to them (Figure 3) [71]. 

Yousif and Thulesius [72] evaluated meloxicam, a preferential COX-2 inhibitor, and indomethacin on the in vitro uterine motility of uterine strips from rats. They found that both drugs reduced the frequency (pre-treatment = 4.5 contractions/min vs. post-treatment = 0.3 contractions/min) and amplitude (pre-treatment = 90 mmHg vs. post-treatment = 10 mm Hg) of contractions in pregnant and non-pregnant animals. These results were corroborated by Rac et al. [71], who analyzed meloxicam’s capacity to inhibit preterm parturition in nine Polled Dorset ewes at 121 days of gestation. After application of a contraction promotor to induce parturition, meloxicam’s action reduced the amplitude and frequency of myometrial contractions (2.25 ± 0.48 contractions per minute and 5.21 ± 0.43 mmHg, respectively), thus inhibiting parturition. The same effect on the amplitude and frequency of contractions has been observed in in vitro studies of the uteri of dairy cattle [73]. The biochemical explanation of this phenomenon lies in the mechanism of action that inhibits the COX-1 and COX-2 enzymes.

Expression of the COX-1 and COX-2 enzymes during gestation increases in uterine tissue, as has been demonstrated in sheep models [5]. Both of these isoforms are fundamental for producing PGs, such as PGF2α and PGE2, which are catalyzed by COX-2 to transform arachidonic acid into PGG2 and final products such as PGF2α, PGE2, and PGH2 by means of peroxides [8,74]. In mice, direct inhibition of COX-2 expression negatively regulates PGF2α synthesis during gestation, triggering the indirect suppression of the expression of c-Fos, which is considered a transcriptional activator of COX-2 [75] that is essential for inducing oxytocin receptors in the myometrium [76]. In a similar study, interference in PG secretion in adult female rats medicated orally with meloxicam at doses of 7.5 and 10 mg/kg from day 20–22 of gestation, prolonged delivery times following a dose-dependent pattern (37–51 h of delay), and produced more stillborn pups (5.3 ± 0.67 fetuses in the 7.5 mg treatment vs. 5.6 ± 0.42 in the dams medicated with 10 mg/kg) [77]. A similar prolongation effect has been seen in dogs when NSAIDs were administered during the final stage of pregnancy [78]. 

The selectivity of the NSAIDs has been associated with both benefits and complications for dams. In dairy cows, for example, administering NSAIDs of the inhibitor of COX-1 type produced adverse effects such as retained placenta, metritis, and culling, because COX-1 is constitutive and participates in diverse physiological functions. In contrast, preferential inhibitors of COX-2 have been related to benefits for the health and productivity of females [79,80]. 

In a study of 237 dairy cows, administration of meloxicam (1 mg/kg) before and after calving had no effect on the health of dams, but increased milk production in 6.8 kg/day more than in the control [81]. Similarly, in Holstein cows, those that were treated with meloxicam had increased milk compared to a control group. There were other benefits with the probability of the medicated cows suffering subclinical mastitis being reduced by 0.05 times, whilst the probability of euthanasia or death was 0.46 times lower [3]. Similar results have been suggested for the use of firocoxib, another selective COX-2 inhibitor [82]. 

The consequences of firocoxib administration for the fetus, compared to meloxicam or flunixin meglumine, was analyzed in the embryos of 30 mares. In that study, Okada et al. [83] evaluated embryonic movement using transrectal ultrasonography every 5 min for 1 h before treatment. Firocoxib did not interfere with embryo mobility at 12 days after pregnancy confirmation, with the embryos maintaining constant movements (measured in movements/hour) before, immediately after, and 24 h after treatment. Contrarily, embryo mobility decreased with the use of flunixin meglumine (pre-treatment = 5.9 ± 0.3; post-treatment = 1.9 ± 0.3) and meloxicam (pre-treatment = 5.8 ± 0.5; post-treatment = 2.3 ± 0.5 movements/h). In contrast, in a study of ewes, meloxicam increased fetal blood flow, albeit with no effect on osmolarity, uterine blood pressure, or maternal–fetal gas exchange [71]. Nevertheless, firocoxib is likely the drug of choice for administration to gestating animals and has been considered the best non-steroidal option for treating pathologies such as endometritis (inflammation of the endometrium) in the early stages of gestation [83]. 

One drug that has been shown to have a harmful effect on fetal viability is flunixin meglumine. Newby et al. [84] studied 34 Holstein cows treated with flunixin and 38 with a placebo, before and after calving. The results showed that the offspring of the animals treated with the flunixin 24 h before parturition had higher mortality rates and an increased probability of placental retention and fever, coupled with lower milk production and a higher risk of metritis development. These effects were attributed to the drug’s property of inhibiting endogenous production of PG by as much as 80% (at a dose of 2.2 mg kg^−1^ for 10 days). Therefore, if the level of PG required to promote contractions of the smooth uterine muscle is reduced, myometrial motility also decreases, and the response of oxytocin is greater, as are the adverse effects [85]. 

In summary, administration of NSAIDs during parturition highlights the selective influence of isoforms on COX with COX-2 selective agents being preferential. The decrease in PG concentration and the tocolytic effect of these analgesics, which are associated with risks for the offspring of some species, have shown no risk to others. Although they are contraindicated for use in gestating females or during dystocia births in rodents, in some instances [86], these drugs have also been proposed for some species as options that do not affect dams or their neonates either biologically or in terms of productivity. 

## 5. Local Analgesics

Local analgesics (LA) are considered the gold standard for controlling acute pain [87,88], especially during parturition in both, animals and humans [7]. Lidocaine, bupivacaine, mepivacaine, and ropivacaine are the most commonly used [89]. Their mechanism of action is through the reversible blockade of voltage-gated Na2+ channels that impedes the transduction of harmful signals and their ensuing transmission [90]. As occurs with sensitive signals, when LA are administered motor signals are also inhibited, generating a decrease in the contraction of both skeletal and smooth muscle, as numerous studies have shown [13,91,92]. To date, however, it is unclear whether these drugs could affect the performance of mothers during labor. In a study of humans, Qian et al. [14] evaluated 213 pregnant women under different pain control treatments (levobupivacaine, ropivacaine, and controls). They found that levobupivacaine did not impede the normal progress of labor, while both levobupivacaine and ropivacaine efficaciously controlled pain and helped maintain all physiological functions intact.

Studies in non-human animals have demonstrated that, like opioids, LA present tocolytic activity in experimental models. A study by Karsli et al. [93] compared the effect of opioids (meperidine, alfentanil, remifentanil) to those of LA (mepivacaine, ropivacaine, bupivacaine) in cell cultures of gestating rats. In those tests, the amplitude and frequency of the contraction of uterine cells decreased significantly with the use of both groups of drugs [4]. The reduction in the number of contractions of the smooth uterine after administration of LA is dose dependent, and the degree of muscular relaxation that the analgesics cause increases as the dosage is increased. This was pointed out by Arici et al. [94] in their evaluation of the effect of 10 accumulated doses of (−8) to 10 (−4) mol/L of mepivacaine, ropivacaine, and bupivacaine on strips of uterine muscle isolated from female Wistar rats at 18–21 days of gestation. Upon stimulating the uterine tissue with oxytocin, both bupivacaine and ropivacaine reduced uterine contractions in a dose-dependent pattern, but mepivacaine exerted the opposite effect by significantly increasing the number of contractions. These results were similar to those published by Li et al. [92], who used full-thick myometrial strips of gestating and non-gestating rats, exposed to cumulative amounts of levobupivacaine and bupivacaine (10-8 mol/L to 10-4 mol/L). Those authors found that at higher amounts both LA caused inhibition of contractibility, with levobupivacaine increasing the amplitude of contractions and bupivacaine increasing their frequency.

The uterine response to progressively greater doses of procaine, lidocaine, or ropivacaine—from 0.1 mg/mL to 0.5 mg/mL and then to 1 mg/mL—was studied using an experimental pig model. In general, the LA increased intrauterine pressure in the isthmus and corpus that was dose dependent, although the authors also observed a reduction in the frequency and amplitude of contractions [95]. These results support the proposal that the reduction in contractibility does not necessarily have a negative effect on performance during parturition but may help regulating the strength of contractions and make them more effective, with the additional advantage of no nociceptive response. 

On the other hand, the muscular relaxation effect of local analgesics leads to a vasodilation effect at the systemic level that reduces blood flow to various systems [96]. This event was observed in a series of studies in pregnant ewes, where epidural administration of LA reduced uterine blood flow by as much as 65 ± 9 mmHg. The consequent reduced fetal blood flow and fetal gas exchange [97] was similar to that observed with opioids, but with the advantage that no mental state or hypnotic effect was induced in the offspring. By the same token, administering epidural analgesia with LA blocks the Ferguson reflex and reduces blood oxytocin level, altering the main beneficial effects of this hormone. These include the promotion of social interaction, formation of the mother–offspring bond, and reduced anxiety, pain, and stress. Based on the fact that LA interrupt the full functioning of the neural pathways, the adverse effects of their use during parturition are attributed to those characteristics [98]. 

Similarly to opioids and NSAIDs, LA can reduce uterine contractions due to their tocolytic action. That reduction has an inverse relation to the strength of the contractions, making them more effective and opening the possibility of improving the performance of dams during parturition. However, the hypotension they cause is an element that must be considered when deciding whether to use these drugs to avoid risks for the fetus and the mother [99]. A summary of the discussed drugs and their effect or benefits during parturition can be seen in Table 1. 

## 6. Perspectives

**Figure 4:**
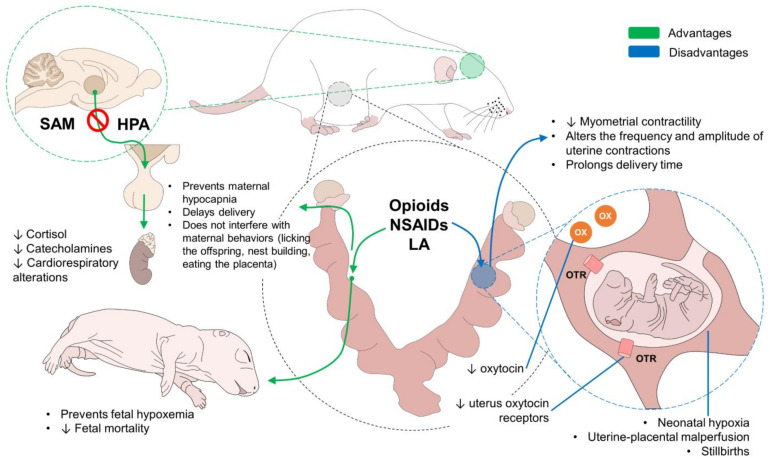
Summary of advantages and disadvantages of analgesia in parturition. Opioids, NSAIDs, and LA can have beneficial or detrimental effects on the mother and the newborn. However, since these actions differ between species and individuals, an appropriate analgesic protocol must consider these factors from a pharmacological point of view. Advantages such as the prevention of hypocapnia and hypoxemia in the dam and the offspring, respectively, are marked in green inside the figure. Disadvantages such as a decrease in OX and OTR are marked with blue. HPA: hypothalamus–pituitary–adrenal axis; LA: local analgesics; NSAIDs: non-steroidal anti-inflammatory drugs; OTR: oxytocin receptors; OX: oxytocin; SAM: sympathetic–adreno–medullary axis.

The control of pain in animals is fundamental for preserving their welfare [1]. The negative impact of pain perception on states of health, hemodynamic stability, the immune system, and the capacity to response to infections are justifications for preventing this process in animals [100,101,102]. The pain produced during physiological events such as parturition requires additional studies that deepen and broaden existing perspectives. This is especially so given the controversy around this topic with some authors supporting the proposition that the presence of pain sustains uterine contractions, whereas others emphasize the potentially harmful effects of the processes triggered when perception of painful sensations is prolonged, the latter increasing the possibility of dystocic births with severe consequences for both mother and fetus (Figure 4) [16].

The analgesics currently utilized during labor have shown an ability to inhibit the activity of the myometrium [4]. This is an obstacle for establishing a balanced analgesic strategy that would prevent pain but also avoid interfering with the normal course of parturition. On the one hand, additional studies are needed to substantiate the idea and importance of pain management and its potential benefits in relation to production. On the other, it is necessary to determine whether the inhibition of uterine contractions has a negative effect on fetal blood flow and causes fetal asphyxia or could improve the effectiveness of contractions and thus enhance performance during parturition [82,103,104]. One option for achieving these objectives (i.e., preventing pain without affecting fetal blood flow or maternal performance) could entail using alternative drugs such as firocoxib, ketamine, cannabinoids, or gabapentinoids, which help inhibit the perception of pain through distinct mechanisms without affecting uterine activity in such a potentially stressful event as parturition [82,103,104,105,106].

## 7. Conclusions

Pain has a dual role during parturition, as the positive feedback to myometrial contractions is essential for expulsion of the fetus, but at the same time, this harmful stimulus may trigger serious physiological consequences. Most of the analgesics mentioned herein have a tocolytic effect, but if clinicians understand the adverse effects that may occur when establishing an analgesic protocol, this effect should not impede managing pain in specific events. Opioids indirectly inhibit uterine contractions. Research has shown that their hypnotic and depressant effects can affect birth performance and offspring vitality. In contrast, the indirect tocolytic effect of NSAIDs may not exceed therapeutic efficacy in maintaining the productive performance of animals after parturition. Finally, the local analgesics considered the gold standard for controlling pain during labor have been shown to decrease the number of contractions but increase their strength, thus facilitating labor. Therefore, the aim of developing a pain management protocol should be to reduce the physiological consequences of these effects. Their administration, however, requires constant monitoring of the course of labor to identify the moment at which the physiological benefit of pain is lost, or when the benefits that analgesia provides during parturition could begin to have detrimental effects on the animals involved.

## Figures and Tables

**Table 1:** Summary of the different analgesic drugs used during parturition in domestic animals.

Drug	Action Mechanism	Effect	Benefit	Species	References
Opioids Morphine Fentanyl Tramadol	Agonism to µ, κ, and δ opioid receptors in the cerebral cortex and spinal cord.	Therapeutic: Systemic analgesia. In myometrium: Decreases the amplitude and frequency of uterine contractions.	Modulates and reduces pain perception.	Bovine, ovine, caprine, equid, and leporids.	[4,50,51,66]
Non-steroidal anti-inflammatory drugs (NSAIDs) Meloxicam Carprofen Firocoxib Flunixin meglumine	COX inhibitor and nociceptive modulator.	Therapeutic: Systemic analgesia. In myometrium: Tocolytic effect. Diminishes the amplitude and frequency of uterine contractions due to a reduction in PG secretion.	Inhibits the production of COX and proinflammatory mediators.	Bovine, equid, and ovine.	[71,72,83]
Local Analgesics Lidocaine Bupivacaine Ropivacaine Levobupivacaine	Sodium channel blockers. Interferes with the transduction and transmission of the nociceptive stimuli.	Therapeutic: Local analgesia. In myometrium: Reduces the amplitude and number of uterine contractions.	Inhibits the transduction and transmission of pain.	Bovine, equid, and pig.	[13,14,93,94]

COX: cyclooxygenases; PG: prostaglandins.

## Data Availability

Not applicable.
